# Seed Priming with Carrot Extract Improves Early Physiological Responses to Salinity in Rice

**DOI:** 10.3390/plants15071082

**Published:** 2026-04-01

**Authors:** Sheila Bigolin Teixeira, Fernanda Reolon de Souza, Stefânia Nunes Pires, Gabriele Espinel Avila, Cristiane Deuner, Geri Eduardo Meneghello, Sidnei Deuner

**Affiliations:** 1 Department of Botany, Federal University of Pelotas, Pelotas 96010-610, RS, Brazil; sheilabigolin@gmail.com (S.B.T.); stefanianunespires@gmail.com (S.N.P.); gabriele.esp@gmail.com (G.E.A.); sdeuner@yahoo.com.br (S.D.); 2 Department of Plant and Soil Sciences, Mississippi State University, Starkville, MS 37962, USA; 3 Faculdade de Agronomia Eliseu Maciel, Federal University of Pelotas, Pelotas 96010-900, RS, Brazil; cdeuner@yahoo.com.br (C.D.); geri_meneghello@hotmail.com (G.E.M.)

**Keywords:** *Oryza sativa* L., *Daucus carota* L., salinity, root morphology, antioxidant activity

## Abstract

Soil salinization is a major constraint on irrigated rice cultivation, mainly due to poor irrigation management and cropping in coastal areas. Seed priming is widely recognized as a cost-effective and practical approach to enhance early growth and improve tolerance to abiotic stresses, including salinity. This study evaluated the effects of seed priming of rice seeds from two cultivars, BRS Querência (Indica) and BRS 358 (Japonica), using aqueous carrot root extract at 0% (water), 25%, and 50% concentrations for 48 h. Seeds were sown in rhizotrons and exposed to 0, 75, or 150 mM NaCl. Morphological, physiological, and biochemical traits were evaluated at 21 days after sowing. Seed priming with carrot extract was associated with improved growth and physiological responses under salinity stress. Under 150 mM NaCl, primed seedlings showed approximately 40% higher chlorophyll index, 35% greater root volume, and 30% higher shoot dry mass compared to unprimed controls. The 25% extract concentration was particularly effective for BRS Querência, which showed enhanced root elongation and a higher nitrogen balance index. Activities of superoxide dismutase, ascorbate peroxidase, and catalase increased by 45–70%, while hydrogen peroxide and malondialdehyde levels decreased by approximately 50%, suggesting enhanced antioxidant responses and improved redox balance. Anthocyanin accumulation also increased in specific cultivar–treatment combinations, suggesting a potential effect on secondary metabolism and antioxidant pathways. Overall, carrot-based seed priming was associated with improved seedling performance, pigment stability, and regulation of oxidative stress under saline conditions. These results suggest that carrot-based seed priming may improve physiological performance under salinity stress.

## 1. Introduction

Rice sustains more than half of the world’s population and remains indispensable for global food and economic security. In the coming decades, as the global population approaches 9 billion, each hectare of arable land will need to produce substantially higher yields than current levels to ensure food supply stability [1,2]. According to Van Ittersum et al. [3], global cereal demand is expected to increase by ~60% by 2050 relative to 2005–2007, with some regions facing even greater pressure and heightened food security risks. Therefore, achieving vertical gains in productivity requires more efficient resource use and improved crop management strategies to meet future food demand [2]. Any reduction in rice productivity could have dramatic consequences for global food security [4].

Outside Asia, Brazil stands as the leading rice producer, with an estimated planted area of 1.60 million hectares for the 2024/25 season and a projected production of 10.587 million tons of paddy rice (equivalent to 7.199 million tons of milled rice) in 2023/24. For 2024/25, production is expected to rise to 12.321 million tons of paddy rice [5]. However, Brazilian rice-based systems are increasingly threatened by climate change, as intensifying abiotic stresses, including salinity, extreme temperatures, and reduced light availability, pose significant risks that can cause substantial yield losses over large areas [6].

Among these stressors, salt stress is a major constraint on crop production, significantly impairing rice growth and yield due to the high salinity sensitivity of rice genotypes. Rice is particularly sensitive to salt stress at all developmental stages, especially during seedling and reproductive phases [7]. Under salinity stress, plant growth, tiller number, biomass accumulation, panicle number, spikelets per panicle, grain-filling percentage, and grain yield significantly declined. Recent evidence demonstrates that salinity reduces early vigor, impairs physiological performance, and accelerates yield penalties in rice under both field and controlled conditions [8]. Salt tolerance in rice is a complex trait governed by multiple quantitative trait loci (QTLs). Although numerous QTLs related to saline–alkali tolerance have been identified, further efforts are required to functionally characterize candidate genes and deploy them in breeding salt-tolerant rice varieties [9]. In this context, understanding the physiological mechanisms underlying growth performance under salinity conditions is essential for improving tolerance and enhancing crop productivity [9].

The expansion of soil salinization is driven by interacting factors, including poor-quality irrigation water, inadequate drainage, rising sea levels, and increasing aridity, all of which favor salt accumulation through enhanced evaporation and limited leaching [10]. Under saline conditions, plants experience both osmotic and ionic stress [11]. Excess sodium in the root zone lowers osmotic potential, restricts water uptake, and triggers tissue dehydration [12], whereas prolonged accumulation of ions disrupts Na^+^/K^+^ homeostasis and essential metabolic functions [13]. Both osmotic and ionic components promote the generation of reactive oxygen species (ROS) and membrane peroxidation [14,15], which, if unregulated, can culminate in lipid degradation and cell death [16]. Plants counter these effects through mechanisms including ion homeostasis, osmotic adjustment, antioxidant systems, and hormonal regulation [17], although these defenses may be overwhelmed under severe stress.

Seed priming is a simple, cost-effective pre-sowing technique that can alleviate the detrimental effects of salinity on seed germination and enable faster, more uniform germination under saline conditions [18]. Among priming approaches, seed priming has been shown to increase germination percentage and uniformity under saline and low-water-potential conditions, compared to non-primed seeds [19]. Recent reviews highlight the rapid expansion of biostimulant research across cereal crops, including rice, with increasing emphasis on improving physiological resilience to abiotic stressors [20]. Plant-derived extracts contain bioactive compounds that can modulate physiological processes and protect tissues against oxidative stress [21]. Carrot roots (*Daucus carota* L.) are rich in sugars, vitamins, and β-carotene and other carotenoids with recognized antioxidant and growth-promoting effects [22]. Extracts from carrot roots have been shown to enhance vigor and mitigate ROS accumulation, improving tolerance in seedlings exposed to abiotic stress, including salinity. β-carotene, in particular, has strong antioxidant activity and can quench reactive oxygen species (ROS) and protect chloroplast membranes under stress, suggesting that carrot-derived carotenoids may contribute to improved redox regulation and early seedling performance under salinity [23].

The cultivars BRS Querência and BRS 358 were selected because they are widely cultivated in southern Brazil and represent agronomically important grain types in irrigated rice systems. Both cultivars are known to be sensitive to salt stress during early growth, making them suitable models for evaluating priming strategies under salinity conditions.

In this context, this study aimed to evaluate the effects of seed priming with carrot root extract on early morpho-physiological responses of rice under salinity stress. Although carrot extract contains bioactive compounds with known antioxidant properties, the present study did not aim to isolate specific osmotic or biochemical mechanisms; rather, it assessed the integrated plant response.

## 2. Materials and Methods

### 2.1. Plant Material and Growth Conditions

The experiment was carried out in a greenhouse located at Embrapa Clima Temperado, Terras Baixas Experimental Station, in Capão do Leão, Rio Grande do Sul, Brazil. Greenhouse conditions were maintained at an average temperature of 25 ± 5 °C and a relative humidity typically ranging between 60 and 80%, under a natural photoperiod. Rice seeds from the cultivars BRS Querência (Indica subspecies) and BRS 358 (Japonica subspecies), after disinfestation with 2% sodium hypochlorite for 5 min, were soaked in water (0%), 25%, and 50% carrot extract. Carrot extract solutions were prepared fresh for each replication and incubated with seeds for 48 h to ensure uniform seed hydration during priming.

To obtain the extract, carrot roots were ground in a fruit processor (Mondial Premium model) and diluted in distilled water. The resulting crude extract was considered 100% concentration. For preparing 25% and 50% solutions, the crude extract was diluted with distilled water at 1:3 (*v*/*v*) and 1:1 (*v*/*v*), respectively. Raw carrot extract was analyzed for pH (6.04), osmotic potential (−0.61 MPa), electrical conductivity (3.63 mS cm^−1^), and β-carotene content (0.0352 mg mL^−1^). In addition, total soluble solids (°Brix), total phenolics, and antioxidant capacity (DPPH method) were quantified to better relate physiological responses to extract composition.

After imbibition, sowing was performed in rhizotrons filled with commercial Plantmax substrate, which was initially moistened with water. Rectangular rhizotrons were used, consisting of two ACM (aluminum composite) plates, 35 cm high and 90 cm wide, separated by aluminum channels with a 1.5 cm spacing. The plates were fixed with fasteners. To favor root growth on the underside of the plate, the rhizotrons were supported on easels at a 45° inclination.

Each rhizotron was subdivided into three experimental units using spacers. Each experimental unit consisted of three plants, resulting in a total of 9 plants per treatment combination (cultivar × priming × salinity). Three seeds were sown per experimental unit for each priming treatment (0%, 25%, and 50%). After sowing, saline treatments composed of 0, 75, and 150 mM NaCl (sodium chloride) were established, with three replicates (rhizotrons) per treatment for each cultivar. The experimental design, therefore, included two cultivars, three priming treatments, and three salinity levels, arranged in a completely randomized factorial scheme. A 150 mM NaCl concentration was used as a severe salinity level commonly employed in controlled studies to induce strong yet measurable stress during rice germination and early seedling growth. The 75 mM NaCl level was included as a moderate stress condition commonly used in physiological studies to induce measurable but non-lethal responses during early seedling development. The solutions were applied at two-day intervals, and to prevent salt accumulation on the substrate, irrigation with water was interspersed between applications. The rhizotrons were kept in a greenhouse for 21 days.

### 2.2. Morphological and Physiological Assessments

At 21 days after sowing, images of each experimental unit were captured with a digital camera and analyzed using WinRHIZO Pro software (Regent Instruments, Inc., WinRHIZO Pro, Quebec City, QC, Canada) to determine the number of pixels representing root area (white color) in each image. The application was calibrated to convert the number of white pixels into metric units (cm^2^), based on a 1 cm^2^ reference scale included in each photograph. Using these measurements, the software calculated total root length (cm) and total root volume (cm^3^).

Leaf area and senescent area were measured using ImageJ software (National Institutes of Health—NIH, Bethesda, MD, USA). Chlorophyll (Chl), anthocyanins (Anth), and nitrogen balance index (NBI) were evaluated with a Dualex FORCE-A device (Orsay, France). Readings were performed on the last fully expanded leaf of each plant. Afterward, the plants were collected to determine the dry mass of aerial parts (DMAP) and roots (DMR).

### 2.3. Enzymatic Activity Assays

Enzymatic extracts were obtained by macerating 200 mg of leaf tissue in liquid N_2_ with 50% (*w*/*w*) insoluble polyvinylpolypyrrolidone (PVPP) and extraction buffer (100 mM potassium phosphate, pH 7.8; 0.1 mM EDTA; and 10 mM ascorbic acid). The homogenate was centrifuged at 12,000 rpm for 10 min at 4 °C, and the supernatant was collected for total protein quantification by the Bradford method [24] and for antioxidant enzyme assays (three replicates per treatment).

Superoxide dismutase (SOD) activity was determined using the nitro-blue tetrazolium (NBT) photoinhibition method [14]. The reaction mixture contained 100 mM potassium phosphate buffer (pH 7.8), 0.1 µM EDTA, 14 mM methionine, 2 µM riboflavin, 75 µM NBT, and 0.05 mL of protein extract. The mixture was incubated under white light at room temperature for 10 min, with a dark control as a blank. Absorbance was measured at 560 nm, and one enzymatic unit (EU) was defined as the amount of enzyme required to inhibit 50% of NBT photochemical reduction.

Ascorbate peroxidase (APX) activity was determined according to Nakano and Asada [25]. The reaction medium consisted of 100 mM potassium phosphate buffer (pH 7.0), 0.1 mM hydrogen peroxide (H_2_O_2_), 0.5 mM ascorbic acid, and 50 µL of protein extract. Absorbance at 290 nm was recorded after 2 min, and APX activity was calculated using the molar extinction coefficient of ascorbate (ε = 2.8 mM^−1^ cm^−1^).

Catalase (CAT) activity was determined as described by Azevedo et al. [26]. The reaction mixture contained 100 mM potassium phosphate buffer (pH 7.0), 12.5 mM H_2_O_2_, and 50 µL of protein extract. Absorbance at 240 nm was recorded after 2 min, and CAT activity was calculated using the molar extinction coefficient for H_2_O_2_ (ε = 39.4 mM^−1^ cm^−1^).

To quantify hydrogen peroxide (H_2_O_2_) and lipid peroxidation, 200 mg of leaf tissue were macerated in liquid N_2_ with 20% (*w*/*w*) PVPP and homogenized in 0.1% (*w*/*v*) trichloroacetic acid (TCA). The homogenate was centrifuged at 12,000 rpm for 20 min at 4 °C. H_2_O_2_ content was determined according to Sinha et al. [27], using a reaction medium containing 10 mM potassium phosphate buffer (pH 7.0), 1 M potassium iodide, and 300 µL of the plant extract. Absorbance was read at 390 nm and compared against a standard H_2_O_2_ calibration curve.

Lipid peroxidation was estimated following the protocol of Heath and Packer [28], based on the thiobarbituric acid (TBA) reaction for quantifying MDA. The reaction mixture contained 0.5% (*w*/*v*) thiobarbituric acid (TBA) and 10% (*w*/*v*) TCA. Samples were incubated at 95 °C for 30 min, cooled in an ice bath, and absorbance was measured at 535 and 600 nm. Lipid peroxidation levels were expressed as nmol malondialdehyde (MDA) using an extinction coefficient of 1.55 mM^−1^ cm^−1^.

### 2.4. Data Collection and Analysis

The experimental design was completely randomized in a 3 × 3 factorial scheme, with factor A representing the soaking treatments and factor B representing the saline treatments. The data were subjected to analysis of variance (*p* ≤ 0.05), and means were compared using Tukey’s test at a 5% significance level with the Assistat software, version 7.7 beta; Federal University of Campina Grande, Campina Grande, Brazil) [15].

## 3. Results

### 3.1. Growth Parameters and Leaf Pigments

**Figure 1:**
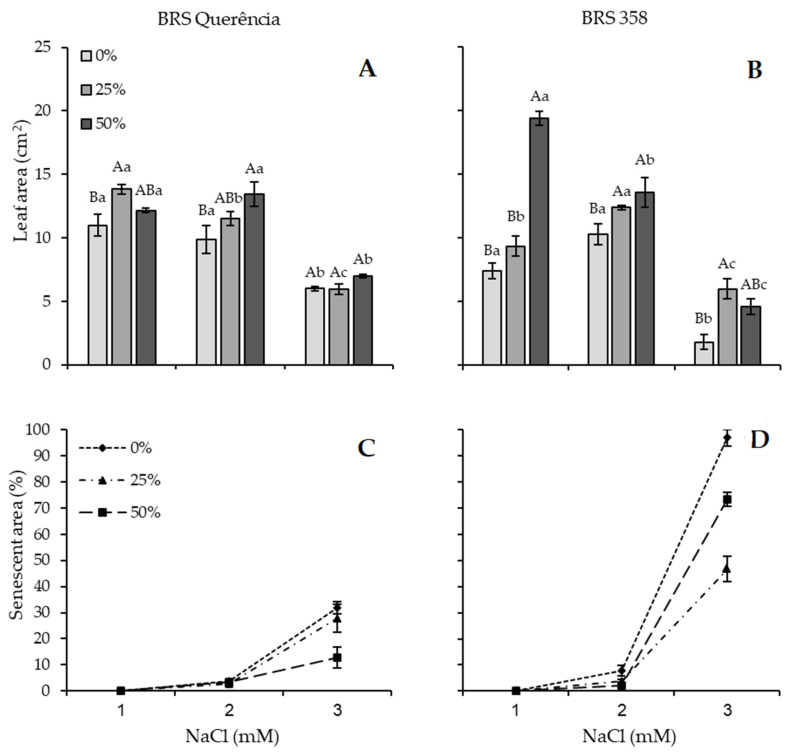
Leaf area (**A**,**B**) and senescent leaf area (**C**,**D**) of rice cultivars BRS Querência and BRS 358 under NaCl treatments (0, 75, 150 mM) and carrot extract seed priming concentrations (0%, 25%, 50%). Different colors (**A**,**B**) and symbol types (**C**,**D**) represent carrot extract concentrations. Means followed by the same letters did not differ from each other by Tukey’s test (*p* < 0.05). Capital letters compare seed imbibition treatments for each salinity level, and lowercase letters compare each seed imbibition treatment between different salinity levels. Bars represent the standard error of the mean of three replicates.

A significant interaction was found between the variables analyzed (*p* < 0.01). Carrot extract was associated with changes in leaf area and senescent leaf area across salinity treatments (Figure 1). For cv. BRS Querência, carrot extract (25 and 50%) increased the leaf area in the 0 and 75 mM NaCl treatments (Figure 1A). In the 150 mM NaCl treatment, although the extracts did not result in a significant effect on the leaf area, an apparent reduction in senescent leaf area was observed, where the absence of carrot extract resulted in 32% of senescent area, compared to only 12.8% when soaked in 50% extract (Figure 1C).

In cv. BRS 358 across salinity levels, carrot extract significantly increased leaf area, with 50% extract showing the greatest effect at 0 and 75 mM NaCl, and 25% extract being most effective at 150 mM NaCl (Figure 1B). Regarding senescence, at the highest saline concentration, the senescent area reached nearly 95% in the absence of carrot extract (Figure 1D).

Seed priming with carrot extract was associated with increases in pigment indices and nitrogen balance in rice plants. In cv. BRS Querência, there was a significant increase in the chlorophyll index in response to carrot extract for all saline treatments and, for the nitrogen balance index, only in the 0 and 75 mM NaCl treatments. The anthocyanin index was higher at the 150 mM NaCl concentration in response to carrot extract (Table 1).

In cv. BRS 358, carrot extract also significantly increased chlorophyll and nitrogen balance indices under saline stress, with the strongest relative improvements observed at 150 mM NaCl when comparing 0% and 50% extract (Table 2). Regarding anthocyanins, the behavior of cv. BRS 358 was somewhat different from that observed in cv. BRS Querência, with a significant reduction in this parameter occurring in the absence of carrot extract and at a 25% concentration in response to saline stress. However, anthocyanin levels remained stable under salinity in plants derived from seeds soaked in 50% carrot extract.

**Figure 2:**
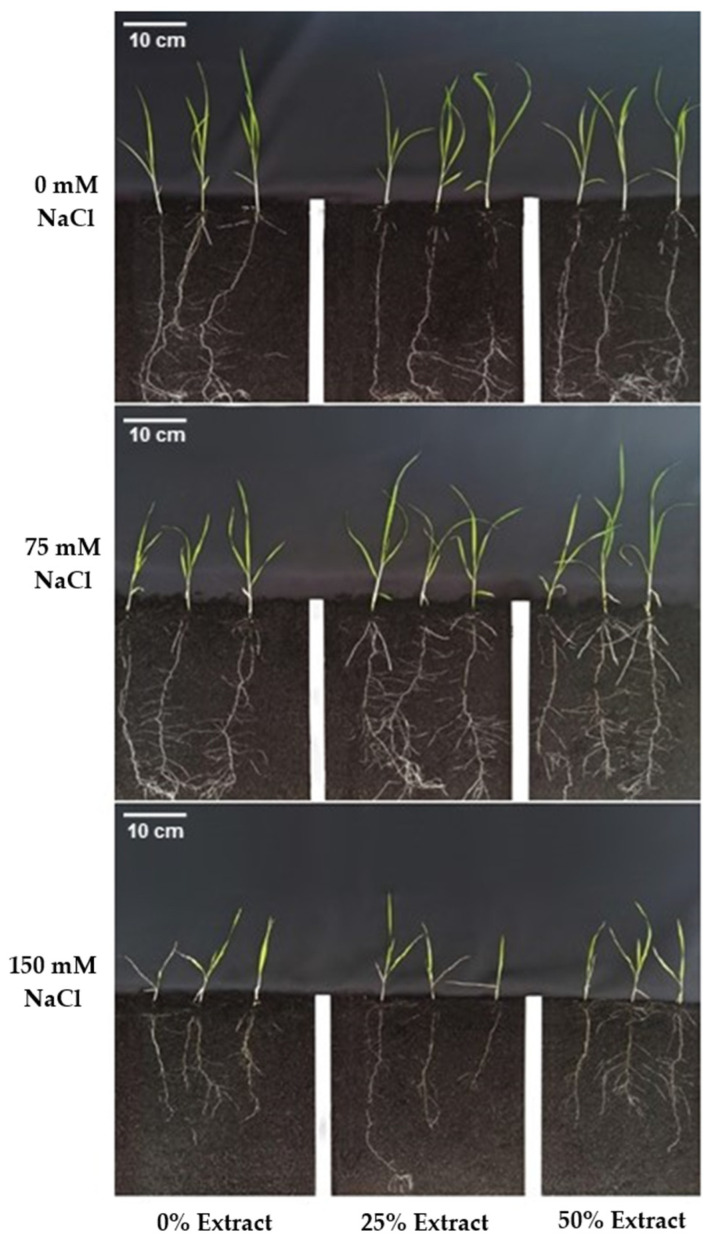
Images of rice plants of cv. BRS Querência at 21 days after sowing, showing the effects of seed osmopriming with carrot extract (0%, 25%, and 50%) under three salinity levels (0, 75, and 150 mM NaCl). Each panel illustrates shoot development and root system architecture under the combined influence of imbibition treatment and saline conditions.

Regarding growth parameters in cv. BRS Querência primed with carrot extract resulted in significantly higher shoot dry mass and total root length in treatments 0 (absence of salinity) and 75 mM NaCl (Figure 2, Table 3). The highest increase was at the 25% extract concentration without salinity, reaching 127.1 cm of total root length, 48.7 cm greater than the control. For root dry mass, a significant difference between carrot extract and salinity was observed only in the 150 mM NaCl treatment, with a lower value at the 25% extract concentration. Root volume in the 75 and 150 mM salinity treatments showed no variation; however, in the absence of salinity, 25% carrot extract produced the greatest increase, consistent with the results for total root length.

**Figure 3:**
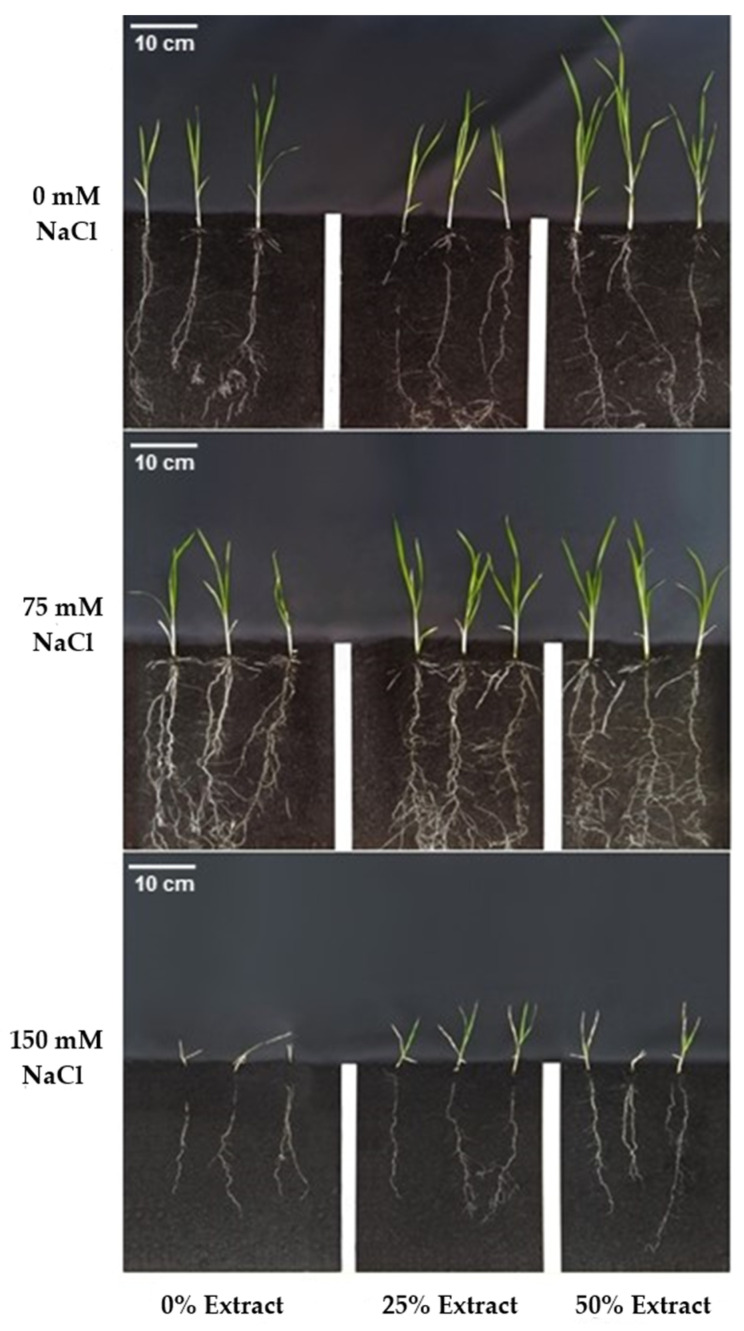
Images of rice plants of cv. BRS 358 at 21 days after sowing, showing the effects of seed osmopriming with carrot extract (0%, 25%, and 50%) under three salinity levels (0, 75, and 150 mM NaCl). Each panel illustrates shoot development and root system architecture resulting from the interaction between imbibition treatment and saline conditions.

In cv. BRS 358, with 50% carrot extract, increased shoot length and shoot dry mass at 0 mM NaCl, reaching 4.7 cm and 30 mg, which were higher than those with 25% extract (Figure 3, Table 4). Although higher salinity reduced growth, the 25% extract treatment at 150 mM NaCl showed a higher SDM than the others (Figure 3). However, MSPA at 150 mM NaCl showed no difference between soaking treatments.

An increase in total root length was observed with the use of carrot extract (25% and 50%) in treatments with 0 mM NaCl. At 75 mM, the highest TRL occurred for this cultivar, associated with 25% extract. At 150 mM NaCl, this variable decreased considerably, but there was no significant difference between the treatments. Regarding root dry mass, it only showed a response to salinity, decreasing significantly at 150 mM NaCl.

The root volume variable was lowest at 150 mM NaCl but increased with the addition of carrot extract. At 150 mM, using 50% extract resulted in a 33% rise in RV compared to 0%, showing a result similar to that in the control (0 mM).

The use of 75 mM NaCl caused a decrease in the average root diameter, which increased significantly at 150 mM. The highest value was observed at a 50% extract, and the lowest at 0% (Table 4).

### 3.2. Enzyme Activity

**Figure 4:**
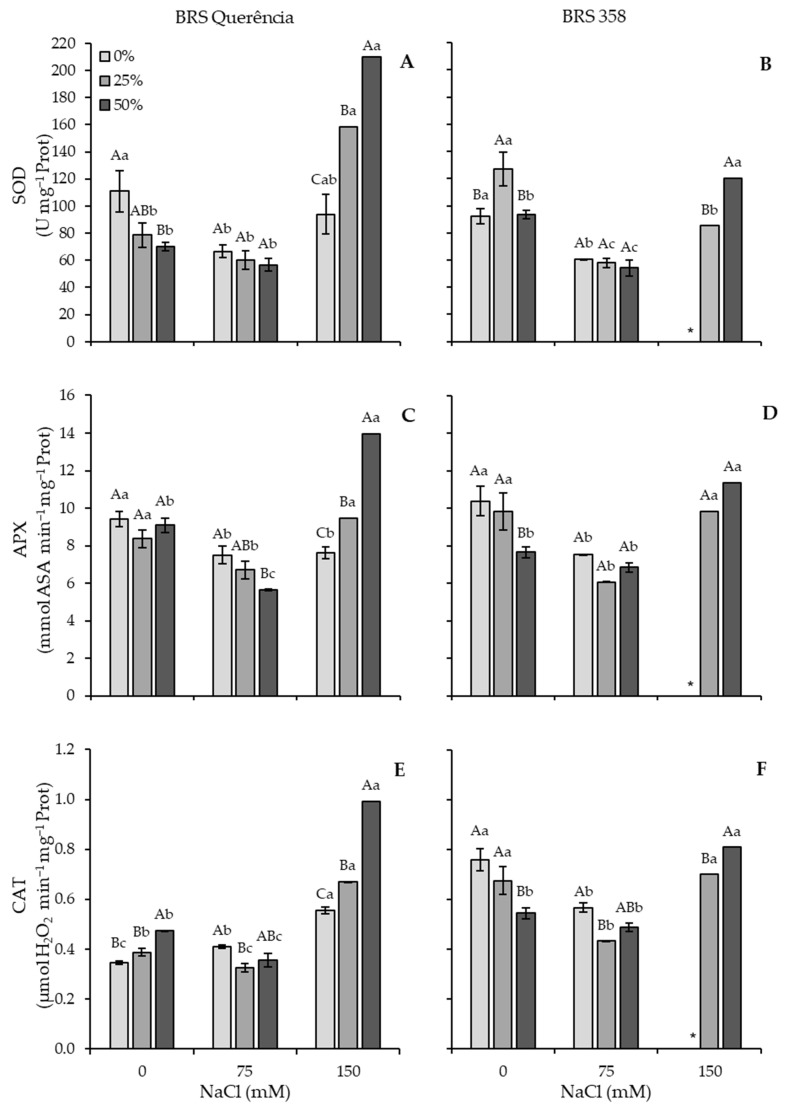
Activity of enzymes superoxide dismutase (**A**,**B**), ascorbate peroxidase (**C**,**D**), and catalase (**E**,**F**) in leaves of rice plants of cvs. BRS Querência (**left**) and BRS 358 (**right**) grown under different percentages of carrot extract and NaCl. Means followed by different capital letters within each salt concentration and lowercase letters between salt concentrations differ from each other by Tukey’s test (*p* < 0.05). * Not evaluated due to high plant biomass suppression.

The 150 mM NaCl treatment significantly increased the activity of all enzymes, with a more pronounced increase in plants treated with carrot extract. Only in BRS 358 were these increases in activity less pronounced than in the 0 mM control, and enzyme activity remained reduced at 75 mM NaCl (Figure 4).

For cv. BRS Querência, the SOD enzyme showed increases of 101.7% with 25% extract and 199.4% with 50% extract from 0 to 150 mM NaCl. The water-soaked treatment (0%) did not differ significantly in enzyme activity (Figure 4A), with the highest activity at 0 mM and the lowest at 150 mM NaCl. In cv. In BRS 358, plants treated with 50% extract exhibited increased enzyme activity compared to the control (0 mM NaCl), showing a 28.6% increase. In contrast, plants with 25% extract showed a 33% decrease, but demonstrated a 47.1% increase from 75 to 150 mM NaCl. The 0% extract treatment was not evaluated due to the suppression of plant biomass caused by high salinity (Figure 4B).

The APX enzyme in cv. BRS Querência decreased in plants with 0% extract and increased by 53.8% in plants with 50% extract from 0 mM to 150 mM NaCl (Figure 4C). In BRS 358, plants with 50% extract showed a 48.4% increase for this enzyme (Figure 4D). The 25% extract treatment did not significantly affect APX activity in either cultivar; however, it showed the lowest activity at 75 mM NaCl. For the CAT enzyme of cv. BRS Querência, activity increased by 60.3%, 73.7%, and 110% from 0 mM to 150 mM NaCl in plants with 0%, 25%, and 50% extract, respectively (Figure 4E). Meanwhile, cv. BRS 358 only showed an increase in plants with 50% extract, while activity decreased at 75 mM NaCl in plants with 0% and 25% extract (Figure 4F).

**Figure 5:**
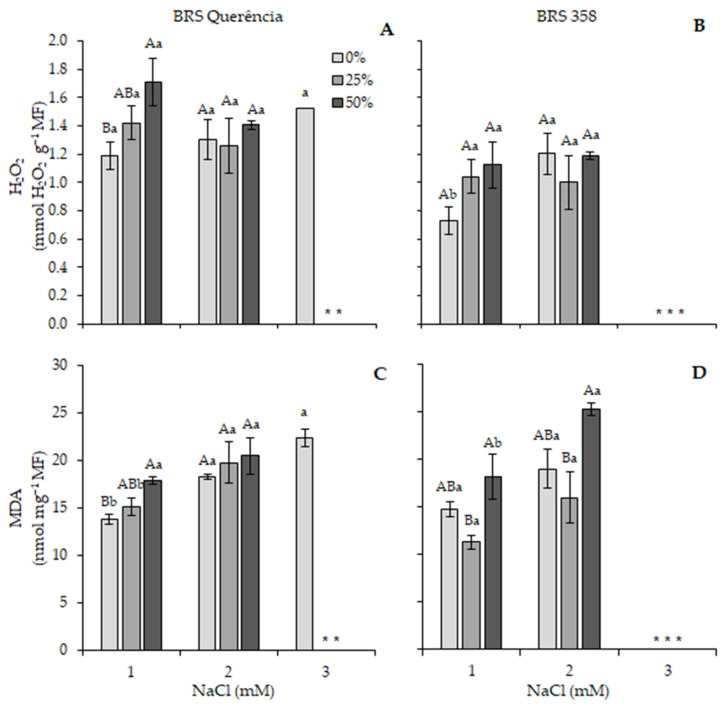
H_2_O_2_ content (**A**,**B**) and lipid peroxidation (**C**,**D**) in the leaves of rice plants of cvs. BRS Querência (**left**) and BRS 358 (**right**) grown under different percentages of carrot extract and NaCl. Means followed by different capital letters within each salt concentration and lowercase letters between salt concentrations differ significantly according to Tukey’s test (*p* < 0.05). * Not evaluated due to high plant biomass suppression. Multiple asterisks (**, ***) indicate the number of non-evaluated bars within each treatment.

Due to the substantial reduction in plant biomass observed at 150 mM, H_2_O_2_ content and lipid peroxidation (MDA) were only measured at 0 and 75 mM NaCl (Figure 5). Hydrogen peroxide levels did not differ significantly from the control in any of the imbibition treatments for cv. BRS Querência. In BRS 358, there was only an increase in H2O2 content in plants imbibed in water (0%), showing a 64.7% rise (Figure 5A,B).

Lipid peroxidation in cv. BRS Querência increased by 33.1% and 30.6% in treatments with 0% and 25% extract, respectively; there was no significant difference in plants with 50% extract (Figure 5C). In BRS 358, a significant increase of 39.1% was observed in the treatment with 50% extract (Figure 5D).

## 4. Discussion

Saline stress affects membrane permeability and impairs plant water and nutrient uptake due to excessive soil salinity. These changes lead to osmotic and ionic imbalances that disrupt cellular homeostasis and induce oxidative stress responses. Stress-induced signaling pathways associated with protein modification and cellular regulation have been implicated in plant adaptation to saline environments [29]. In this study, increased soil salinity was linked to a reduction in plant leaf area and a corresponding increase in senescent area (Figure 1). These effects are consistent with morphological adjustments typically observed under physiological drought, in which plants alter leaf anatomy to reduce transpiration and sodium uptake, a common adaptive response reported under stress conditions [30].

Salt stress significantly reduced growth attributes in both cultivars, particularly at 150 mM NaCl (Table 3 and Table 4). Das et al. [31] observed similar results in 15 rice genotypes, with decreased seedling height, leaf area, and biomass, accompanied by increased leaf curling and drying. These effects arise mainly from reduced cell turgor induced by ion toxicity. Shoot elongation was more affected than root length, which is typical of osmotic stress, since roots tend to elongate to explore deeper soil layers for water (Table 3 and Table 4). In the present study, mild salinity was also reported to initially stimulate root growth, as seen in cv. BRS 358 at 75 mM NaCl (Table 4). Conversely, higher levels (150 mM) caused a sharp decline in root length and volume, consistent with reduced water and nutrient uptake [32]. Recent studies indicate that salt-induced growth and physiological impairments in rice are closely associated with disrupted Na^+^ and K^+^ homeostasis, leading to elevated Na^+^/K^+^ ratios and enhanced reactive oxygen species accumulation under saline conditions [33]. Although Na^+^ and K^+^ contents were not quantified in the present study, several of the evaluated physiological attributes, including chlorophyll index, nitrogen balance index, antioxidant enzyme activity, H_2_O_2_ accumulation, and MDA levels, are widely used as indirect indicators of ionic imbalance and disrupted Na^+^/K^+^ homeostasis under saline conditions. These patterns align with recent evidence showing that oxidative stress and ion regulation are tightly coordinated during salt exposure in rice, with alterations in ROS metabolism often reflecting underlying shifts in Na^+^ and K^+^ distribution [11]. Although these parameters are widely used as indirect indicators, they do not replace direct quantification of ion homeostasis, which should be addressed in future studies.

Similar physiological patterns were previously documented by Teixeira et al. [34], who demonstrated that conditioning rice seeds with plant extracts, particularly carrot extract, enhanced early vigor and mitigated the effects of abiotic stress during germination and seedling establishment. These earlier findings support the interpretation that biostimulant-based seed conditioning may contribute to early physiological adjustments associated with improved stress responses, reinforcing the responses observed in the present study.

Because our experimental design prioritized early physiological responses to seed priming, ion profiling (e.g., via ICP-OES) was beyond the study’s scope. However, the present findings provide a basis for future studies integrating Na^+^/K^+^ quantification and correlation analyses to more comprehensively elucidate the mechanisms by which carrot extract enhances salt tolerance.

As observed for shoot and root length, shoot and root dry mass also decreased with increasing salinity, as both variables are closely correlated [35]. Khare et al. [36] attributed these reductions mainly to Na^+^ and Cl^−^ accumulation in tissues. Similar results have been reported for rice [31] and cowpea [37], both of which show declines in leaf area and biomass under salinity. Yamazaki et al. [38] demonstrated that biomass production under saline conditions (0, 50, 100, and 150 mM NaCl) varied widely among sorghum accessions and was used as an indicator of salt tolerance to identify tolerant genotypes for breeding purposes. Consistently, seed conditioning studies with carrot extract have demonstrated improvements in early vigor and seedling robustness under stress conditions [34], reinforcing the potential of plant-based extracts to enhance morpho-physiological performance during early establishment. In addition, the increase in average root diameter observed here likely reflects a reduction in the number of secondary roots, with predominance of thicker primary roots under salt stress (Table 3 and Table 4).

The application of carrot extract was associated with improved plant growth, supporting its potential as a biostimulant under the conditions evaluated. Extracts from various plant materials are known to contain growth-promoting substances such as amino acids, phytohormones, and phenolic compounds that enhance cell division and elongation [20]. Puchooa and Ramburn [22] reported similar findings in *Daucus carota* explants, where carrot juice increased biomass and moisture content. Likewise, Abbas and Akladious [23] demonstrated that carrot extract mitigated salt stress in cowpea by regulating physiological and antioxidant responses. The observed responses may be partially attributable to the bioactive composition of carrot extract, including carotenoids and phenolic compounds, as reported in the literature, although the individual contributions of these compounds were not directly evaluated in this study. Extracts from plant materials, including carrot extract, are known to contain bioactive compounds such as amino acids, phytohormones, carotenoids, and phenolics that can promote plant growth and physiological responses [20]. Recent studies have shown that the application of plant growth regulators can mitigate the adverse effects of salinity on crop growth and physiological performance [37]. Previous research using carrot extract has shown comparable benefits, including enhanced vigor and improved metabolic stability during early seedling development [34], as well as cytogenetic protection of root meristems under saline stress [39].

Saline stress not only imposes osmotic constraints on plant water status but also reduces photosynthetic pigment content, soluble sugars, and protein levels, while inducing proline accumulation in rice seedlings [40]. Chlorophyll content is widely used as a non-destructive indicator of plant nitrogen status, particularly when assessed together with optical indices derived from leaf pigments [41]. In the present study, chlorophyll indices declined with increasing salinity (Table 1 and Table 2), consistent with previous studies on rice [31] and cowpea [37]. However, in cv. BRS Querência, chlorophyll levels were maintained or even increased under moderate salinity (75 mM NaCl). Salt stress is known to reduce chlorophyll content and photosynthetic performance in rice seedlings, with tolerant genotypes maintaining higher pigment levels under salinity [7]. Similarly, nitrogen-use efficient rice plants exhibit improved chlorophyll retention and physiological performance under saline conditions, which is consistent with the patterns observed in this study [40].

The nitrogen balance index (NBI), defined as the ratio between chlorophyll and flavonoid content, reflects plant nitrogen availability and provides a non-destructive estimate of nitrogen nutrition [41]. Given that approximately 70% of leaf nitrogen is localized in chloroplasts [42], decreases in NBI under high salinity indicate both photosynthetic and nutritional impairment. This relationship reinforces nitrogen’s role in promoting leaf expansion and photosynthetic efficiency [43]. Consistent with these findings, our results support the coordinated regulation of metabolic and photosynthesis-related processes under stress conditions, highlighting the role of redox-mediated signaling in driving physiological adjustments during stress adaptation [44].

Flavonoids, particularly anthocyanins, constitute major non-enzymatic antioxidants that accumulate under stress [45]. In this study, anthocyanin indices tended to increase with salinity, especially at 50% extract, suggesting a potential effect of carrot extract on secondary metabolism. However, this response was genotype-dependent, as no consistent increase was observed in cv. BRS 358. Chutipaijit et al. [46] reported that salinity-tolerant rice varieties accumulated nearly twice the flavonoid content of sensitive ones, which supports our findings.

Exposure to abiotic stress conditions enhances the production of reactive oxygen species (ROS), which can cause oxidative damage to cellular membranes, proteins, and nucleic acids. To mitigate oxidative stress, plants activate enzymatic antioxidant systems, including superoxide dismutase (SOD), ascorbate peroxidase (APX), and catalase (CAT). SOD initiates ROS detoxification by converting superoxide radicals (O_2_**^•−^**) into hydrogen peroxide (H_2_O_2_), which is subsequently scavenged by APX and CAT distributed across multiple subcellular compartments, thereby maintaining cellular redox homeostasis [47]. Recent research demonstrates that hydrogen peroxide functions as a positive modulator of abscisic acid (ABA) signaling through redox-dependent modification of stress-responsive transcription factors, promoting the coordinated activation of antioxidant defense systems under stress conditions [48].

In both cultivars, enzyme activity was maintained or slightly reduced at 75 mM NaCl, but significantly increased at 150 mM NaCl, suggesting a potential contribution of the extract to improved physiological responses under salinity (Figure 4). The magnitude of this increase was proportional to the concentration of carrot extract, suggesting a potential contribution of the extract to improved physiological responses under salinity. The effect was more pronounced in cv. BRS Querência, which displayed higher SOD and CAT activity, is consistent with its superior growth performance. Elevated SOD and CAT activity under salinity has been widely reported in tolerant rice cultivars [49]. These patterns are consistent with ROS detoxification reported in the literature, as SOD-derived H_2_O_2_ is subsequently degraded by CAT [49]. Similarly, higher APX activity in tolerant soybean and rice genotypes supports the same adaptive mechanism [50]. These findings align with the recent studies demonstrating coordinated regulation of ion homeostasis and ROS metabolism under salt stress in rice [44]. Cytogenetic analyses in rice roots have also demonstrated that carrot extract can reduce salt-induced cellular damage, reinforcing its role in stabilizing meristematic activity under oxidative stress [39].

Salt and saline-alkaline stress adversely affect plant growth and development by causing ionic imbalances, osmotic stress, and disruption of cellular homeostasis. Adaptive responses involve coordinated physiological regulation and genetic components that underlie stress tolerance. In rice, several quantitative trait loci (QTLs), including the Saltol region associated with Na^+^/K^+^ homeostasis and genes involved in ion transport and vacuolar sequestration, have been identified as key targets for breeding salt-tolerant cultivars [9,51]. In parallel, carotenoid-related pathways have also been implicated in stress adaptation, as increased xanthophyll accumulation derived from β-carotene metabolism has been associated with reduced lipid peroxidation and enhanced tolerance to oxidative stress in rice [52]. These mechanisms may help explain the patterns observed in this study, although direct evidence linking these pathways to the responses measured here was not obtained. The generation of ROS in chloroplasts and mitochondria is a significant indicator of oxidative stress under salinity [50]. Cho and Seo [53] observed increased H_2_O_2_ under cadmium stress in Arabidopsis thaliana due to inhibition of antioxidant enzymes, whereas in rice, reduced H_2_O_2_ levels suggest enhanced APX and CAT activity [54]. Lipid peroxidation (MDA) increased under 75 mM NaCl, particularly in cv. BRS 358, indicating the occurrence of oxidative stress. This aligns with the findings of Demiral and Türkan [54], who reported higher MDA accumulation in salt-sensitive rice genotypes. This perspective aligns with cytogenetic findings demonstrating that carrot extract mitigates chromosomal and meristematic damage under saline conditions [39], reinforcing its potential to enhance cellular resilience during stress. Abbas and Akladious [23] also reported enhanced antioxidant enzyme activity in *Vigna sinensis* treated with carrot extract under salinity, consistent with our results. The observed responses may be partially associated with β-carotene-related pathways described in the literature [52]. Even at low concentrations, bioactive compounds can stimulate antioxidant defense pathways and enhance plant resilience to stress conditions [20]. Natural biostimulants and redox-related signaling molecules have emerged as sustainable strategies to improve stress tolerance by activating transcriptional networks and maintaining cellular redox homeostasis in crops [47]. Although the salinity levels applied in this study do not encompass the full range typically observed in Brazilian rice-growing areas, the objective was not to establish agronomic salinity thresholds for field production but to evaluate early morpho-physiological responses under contrasting stress intensities. The salinity treatments were selected to represent moderate and severe stress conditions commonly used in physiological studies. The inclusion of intermediate salinity levels (25–50 mM NaCl) would be valuable for agronomic refinement and is therefore proposed as an important direction for future research.

It is essential to consider that the controlled substrate used in this study differs from field soils in several physical, chemical, and biological properties, including bulk density, organic matter content, cation exchange capacity, and microbial activity. These factors influence water retention, aeration, and the spatial distribution of salts in the root zone. In irrigated rice systems, salt accumulation is often heterogeneous across soil layers, and this stratification may alter the magnitude of osmotic and ionic stress experienced during early establishment. Because this study was conducted under controlled conditions, the observed responses may not fully represent field scenarios. Future experiments should evaluate its performance under field scenarios, where soil properties, drainage patterns, and microbial interactions may modulate biostimulant efficacy. Seed priming appears to be a practical approach for field application, although additional strategies such as soil drenching or foliar application could be explored. Further studies evaluating growth, ion homeostasis, and yield under different salinity conditions will be necessary to support practical recommendations.

## 5. Conclusions

Salinity markedly impaired rice growth, pigment content, and metabolic stability, leading to reductions in leaf area, biomass, and photosynthetic efficiency. These effects were accompanied by declines in chlorophyll and nitrogen balance indices, reduced enzymatic activity under moderate stress, and increased oxidative damage at higher salinity levels. However, seed priming with carrot root extract consistently attenuated these effects under the controlled conditions evaluated. Plants derived from seeds soaked in 25–50% extract exhibited improved leaf development, higher antioxidant enzyme activity (SOD, CAT, and APX), and lower lipid peroxidation, reflecting enhanced oxidative homeostasis and adaptive plasticity under saline conditions.

The protective effect of carrot extract appears to be related to its rich bioactive compound profile, including β-carotene, vitamins, and phenolics, which are known to participate in redox regulation and antioxidant protection. Between the evaluated cultivars, BRS Querência displayed greater physiological stability and metabolic resilience to salinity than BRS 358, indicating a superior capacity for enzymatic and structural adjustment.

Overall, the results indicate that carrot-based seed priming can improve early physiological responses to salinity by modulating redox balance and antioxidant defenses, although broader agronomic validation is still required. Thus, carrot extract may serve as a complementary biostimulant strategy and should be further explored in long-term, field-based studies to assess its practical relevance for saline rice production systems.

## Figures and Tables

**Table 1:** Chlorophyll index (Chl), nitrogen balance index (NBI), and anthocyanin index (Anth) of rice plants of cv. BRS Querência using different percentages of carrot extract in imbibition and NaCl.

	NaCl (mM)	Carrot Extract (%)						Average	CV (%)
		0		25		50			
Chl	0	7.50 ± 0.35	Bb	14.50 ± 1.49	Aa	14.30 ± 2.00	Aa	12.1	10.9
	75	10.20 ± 0.97	Ba	13.40 ± 0.52	Aa	14.40 ± 1.65	Aa	12.7	
	150	6.60 ± 0.71	Bb	8.60 ± 0.57	ABb	9.10 ± 0.37	Ab	8.1	
	Average	8.10		12.17		12.60		-	
	CV (%)	9.8							
NBI	0	6.90 ± 0.47	Bb	14.5 ± 2.24	Aa	15.6 ± 1.48	Aa	12.3	11.9
	75	10.20 ± 0.90	Ba	14.1 ± 0.83	Aa	16.1 ± 1.57	Aa	13.5	
	150	6.20 ± 0.91	Ab	6.5 ± 1.09	Ab	8.7 ± 1.17	Ab	7.1	
	Average	7.77		11.70		13.47		-	
	CV (%)	11.6							
Anth	0	0.2 ± 0.016	Aa	0.21 ± 0.008	Aab	0.19 ± 0.003	Ab	0.2	13.2
	75	0.19 ± 0.015	Aa	0.18 ± 0.011	Ab	0.18 ± 0.007	Ab	0.18	
	150	0.17 ± 0.008	Ba	0.25 ± 0.049	Aa	0.25 ± 0.024	Aa	0.22	
	Average	0.19		0.21		0.21		-	
	CV (%)	8.00							

Values are presented as mean ± standard deviation (SD). Means followed by the same uppercase letters do not differ among carrot extract concentrations within each NaCl level (Tukey, *p* < 0.05) and means followed by the same lowercase letters do not differ among NaCl levels within each carrot extract concentration (Tukey, *p* < 0.05). CV = coefficient of variation.

**Table 2:** Chlorophyll index (Chl), nitrogen balance index (NBI), and anthocyanin index (Anth) of rice plants of cv. BRS 358 using different percentages of carrot extract in imbibition and NaCl.

	NaCl (mM)	Carrot Extract (%)						Average	CV (%)
		0		25		50			
Chl	0	13.50 ± 1.56	Ba	15.40 ± 0.53	ABa	17.90 ± 1.28	Aa	15.6	11.6
	75	9.40 ± 1.93	Bb	15.81 ± 1.29	Aa	16.60 ± 0.91	Aa	13.9	
	150	2.91 ± 1.58	Bc	4.62 ± 4.12	ABb	7.81 ± 0.76	Ab	5.1	
	Average	8.60		11.90		11.40		-	
	CV (%)	19.5							
NBI	0	12.50 ± 1.39	Ba	14.40 ± 0.98	ABa	18.12 ± 1.59	Aa	15	13.1
	75	9.90 ± 2.79	Ba	16.10 ± 1.28	Aa	17.70 ± 0.83	Aa	14.6	
	150	4.50 ± 1.81	Bb	5.42 ± 4.84	ABb	10.50 ± 1.17	Ab	6.6	
	Average	8.81		12.00		12.40		-	
	CV (%)	22.5							
Anth	0	0.21 ± 0.037	Aa	0.23 ± 0.062	Aa	0.18 ± 0.004	Aa	0.21	26.1
	75	0.14 ± 0.044	ABab	0.1 ± 0.048	Bb	0.19 ± 0.015	Aa	0.14	
	150	0.08 ± 0.031	Bb	0.06 ± 0.051	Bb	0.21 ± 0.044	Aa	0.12	
	Average	0.14		0.12		0.19		-	
	CV (%)	27.80							

Values are presented as mean ± standard deviation (SD). Means followed by the same uppercase letters do not differ among carrot extract concentrations within each NaCl level (Tukey, *p* < 0.05), and means followed by the same lowercase letters do not differ among NaCl levels within each carrot extract concentration (Tukey, *p* < 0.05). CV = coefficient of variation.

**Table 3:** Shoot dry mass (SDM), total root length (TRL), root dry mass (RDM), and total root volume (RV) of rice plants of cv. BRS Querência using different percentages of carrot extract in imbibition and NaCl.

	NaCl (mM)	Carrot Extract (%)						Average	CV (%)
		0		25		50			
SDM (mg)	0	33.87 ± 8.72	Ba	40.76 ± 5.97	ABa	48.5 ± 6.17	Aa	41.1	19
	75	38.72 ± 3.87	Ba	49.61 ± 5.33	Aa	51.5 ± 3.51	Aa	46.6	
	150	14.90 ± 2.43	Ab	15.70 ± 0.68	Ab	15.5 ± 2.06	Ab	15.4	
	Average	29.2		35.4		38.5		-	
	CV (%)	11.1							
TRL (cm)	0	78.37 ± 16.58	Ba	127.12 ± 1.98	Aa	112.63 ± 3.04	Aa	106	14.6
	75	70.59 ± 0.62	Ba	97.34 ± 6.10	Ab	96.89 ± 12.78	Aa	88.3	
	150	41.73 ± 4.91	Ab	44.48 ± 9.22	Ac	44.95 ± 12.36	Ab	43.7	
	Average	63.56		89.65		84.82		-	
	CV (%)	9.6							
RDM (mg)	0	17.071 ± 3.67	Aa	14.85 ± 3.92	Aa	17.15 ± 2.95	Aa	16.7	26
	75	16.93 ± 1.93	Aa	18.15 ± 0.78	Aa	18.70 ± 1.40	Aa	17.9	
	150	9.35 ± 0.38	Ab	6.37 ± 0.27	Bb	8.00 ± 1.00	ABb	7.9	
	Average	14.45		13.10		14.62		-	
	CV (%)	6.5							
RV (cm^3^)	0	0.08 ± 0.0017	Bb	0.14 ± 0.0038	Aa	0.10 ± 0.0148	Ba	0.11	14.4
	75	0.12 ± 0.0103	Aa	0.11 ± 0.0103	Aa	0.12 ± 0.0143	Aa	0.11	
	150	0.05 ± 0.0200	Ac	0.05 ± 0.0058	Ab	0.07 ± 0.0205	Ab	0.06	
	Average	0.08		0.10		0.09		-	
	CV (%)	13.90							

Values are presented as mean ± standard deviation (SD). Means followed by the same uppercase letters do not differ among carrot extract concentrations within each NaCl level (Tukey, *p* < 0.05) and means followed by the same lowercase letters do not differ among NaCl levels within each carrot extract concentration (Tukey, *p* < 0.05). CV = coefficient of variation.

**Table 4:** Shoot dry mass (SDM), total root length (TRL), root dry mass (RDM), and total root volume (RV) of rice plants of cv. BRS 358 using different percentages of carrot extract in imbibition and NaCl.

	NaCl (mM)	Carrot Extract (%)						Average	CV (%)
		0		25		50			
SDM (mg)	0	32.54 ± 4.65	Ba	40.90 ± 2.34	Bb	70.90 ± 4.33	Aa	50.11	6.3
	75	49.81 ± 7.93	Ba	63.75 ± 8.63	Aa	55.62 ± 2.53	ABb	56.4	
	150	5.52 ± 1.09	Ab	11.68 ± 3.24	Ac	7.79 ± 0.97	Ac	8.6	
	Average	31.62		38.76		44.78		-	
	CV (%)	16.2							
TRL (cm)	0	108.30 ± 2.91	Bb	131.8 ± 6.22 Ab	Ab	130.8 ± 13.39	Ab	123.60	6.70
	75	145.40 ± 16.51	Ba	186.70 ± 6.60	Aa	155.60 ± 3.97	Ba	162.53	
	150	47.60 ± 4.51	Ac	46.33 ± 2.96	Ac	53.12 ± 5.71	Ac	49.98	
	Average	100.32		121.71		113.04		-	
	CV (%)	9.80							
RDM (mg)	0	20.07 ± 2.44		21.40 ± 1.80		23.50 ± 0.29		22.00 a	17.8
	75	18.9 ± 1.94		18.13 ± 3.03		19.4 ± 1.20		18.85 a	
	150	4.06 ± 0.94		3.9 ± 1.62		4 ± 0.37		4.00 b	
	Average	14.5		14.5		15.7		-	
	CV (%)	9.5							
RV (cm^3^)	0	0.1 0.0990 ± 0.0127		0.1470 ± 0.0090		0.1320 ± 0.0077		0.13 a	9.4
	75	0.1083 ± 0.0113		0.1723 ± 0.0277		0.1363 ± 0.0026		0.14 a	
	150	0.0563 ± 0.0039		0.0737 ± 0.0029		0.1033 ± 0.0154		0.08 b	
	Average	0.09	B	0.13	A	0.12	A	-	
	CV (%)	17.6							

Values are presented as mean ± standard deviation (SD). Means followed by the same uppercase letters do not differ among carrot extract concentrations within each NaCl level (Tukey, *p* < 0.05), and means followed by the same lowercase letters do not differ among NaCl levels within each carrot extract concentration (Tukey, *p* < 0.05). CV = coefficient of variation.

## Data Availability

The original contributions presented in this study are included in the article. Further inquiries can be directed to the corresponding author.
